# Sex-specific association of serum uric acid trajectories with risk of incident retinal arteriosclerosis in Chinese population: A population-based longitudinal study

**DOI:** 10.3389/fcvm.2023.1116486

**Published:** 2023-02-28

**Authors:** Ruirui Geng, Qinbei Feng, Mengmeng Ji, Yongfei Dong, Shuanshuan Xu, Chunxing Liu, Yufeng He, Zaixiang Tang

**Affiliations:** ^1^Department of Biostatistics, School of Public Health, Suzhou Medical College of Soochow University, Suzhou, China; ^2^Jiangsu Key Laboratory of Preventive and Translational Medicine for Geriatric Diseases, Suzhou Medical College of Soochow University, Suzhou, China; ^3^Department of Laboratory, Hua Dong Sanatorium, Wuxi, China; ^4^Department of Stomatology, Hua Dong Sanatorium, Wuxi, China

**Keywords:** retinal arteriosclerosis, retinal atherosclerosis, fundus arteriosclerosis, SUA trajectories, group-based trajectory modelling

## Abstract

**Background:**

The impact of serum uric acid (SUA) trajectories on the development of retinal arteriosclerosis is uncertain. The purpose of this study was to identify adult SUA trajectories by sex and determine their association with risk of retinal arteriosclerosis.

**Methods:**

In this longitudinal study, 4,324 participants who were aged between 18 and 60 years without retinal arteriosclerosis at or before baseline (from January 1, 2010, through December 31, 2010) were included. Group-based trajectory modeling was used to identify SUA trajectories during the exposure period (from January 1, 2006, through December 31, 2010). Cox proportional-hazards models were applied to evaluate the associations between SUA trajectories and the risk of incident retinal arteriosclerosis during the outcome period (from January 1, 2011, through December 31, 2019).

**Results:**

4 distinct SUA trajectories were identified in both women and men: low, moderate, moderate-high, and high. During a median follow-up of 9.54 years (IQR 9.53–9.56), 97 women and 295 men had developed retinal arteriosclerosis. In the fully adjusted model, a significant association between the moderate-high SUA trajectory group and incidence of retinal arteriosclerosis was observed only in men (HR: 1.76, 95% CI: 1.17–2.65) compared with the low trajectory group, but not in women (HR: 0.77, 95% CI: 0.39–1.52). Also, the high SUA trajectory group had the highest risk with an adjusted HR of 1.81 (95% CI, 1.04–3.17) in men. However, they did not exhibit a substantially increased risk in women.

**Conclusion:**

Higher SUA trajectory groups were significantly associated with an increased risk of incident retinal arteriosclerosis in men but not in women.

## 1. Introduction

Retinal arteriosclerosis/atherosclerosis, including senile degenerative sclerosis and retinal small artery sclerosis, is considered as an indicator of systemic atherosclerotic damage (1). And the assessment of retinal arteriosclerosis is frequently used as a screening for subclinical atherosclerosis in a general population (1). Arterial stiffness with age and can be accelerated by different factors, such as diabetes and hypertension (2, 3). In the most recent studies, growing evidence has shown that the retinal arteriolosclerosis is associated with a range of cardiovascular diseases (CVDs), such as stroke, heart failure, myocardial infarction, coronary heart disease, and other CVDs (4–9). Retinal arteriosclerosis is inevitably accompanied by increased many comorbidities. In addition, with the huge increase in health care costs (4), there is a need to study the predictive factors for early intervention. The retina is also the only deep-seated blood vessel in the body that can be directly visualized in a non-invasive manner (5). Thus, the retinal arterioles offer an opportunity to noninvasively explore the relation of retinal arteriosclerosis to other diseases. This means that early detection of modifiable risk factors for retinal arteriosclerosis is of the utmost importance.

Serum uric acid (SUA), the ultimate metabolite of purines in the body. Accumulating epidemiological and clinical evidence has demonstrated that elevated SUA is associated with a variety of cardiovascular disorders, including hypertension, diabetes, coronary heart disease, metabolic syndrome, etc. (10–17). However, limited research has been performed on the associations of SUA with arteriosclerosis, especially retinal microvessels. And the role of SUA as an independent risk factor in arteriosclerosis incidence is still controversial. Several studies have demonstrated that increased SUA is a significant risk factor for arteriosclerosis (18, 19). Whereas, some studies showed that the association between SUA concentration and atherosclerosis exists only in men and not in women (20, 21), while others suggested that the relationship is much stronger in women than in men (19). Furthermore, there are also a few studies that hold opposing views, which showed that uric acid is a natural peroxynitrite scavenger (22), and is not associated with arteriosclerosis (23, 24), even observed that the increase of SUA is associated with the increase of retinal arteriolar caliber in women (25). A previous population-based cross-sectional study has reported that increased SUA concentrations are risk factors for retinal arteriosclerosis in men but not in women (21). The study only assessed SUA levels at a single time point, and did not consider the potential impact of SUA levels experienced earlier or the changes in SUA levels over time. However, little is known regarding whether long-term SUA trajectory patterns exist and how those patterns relate to retinal arteriosclerosis events. Characteristics of trajectory patterns may help identify populations at high risk for retinal arteriosclerosis events who would benefit from interventions to modify SUA elevation and prevent retinal arteriosclerosis. The role of trajectory in the association between SUA and retinal arteriosclerosis should be examined.

Therefore, the aims of this study were to use a population-based longitudinal cohort database (1) to identify the sex-specific subgroups of individuals with similar trajectories in SUA during a 5-year exposure period and (2) to determine the independent association of these SUA trajectories with the risk of retinal arteriosclerosis during a subsequent long-term follow-up period among adults without hypertension and diabetes.

## 2. Materials and methods

### 2.1. Study design and participants

**Figure 1:**
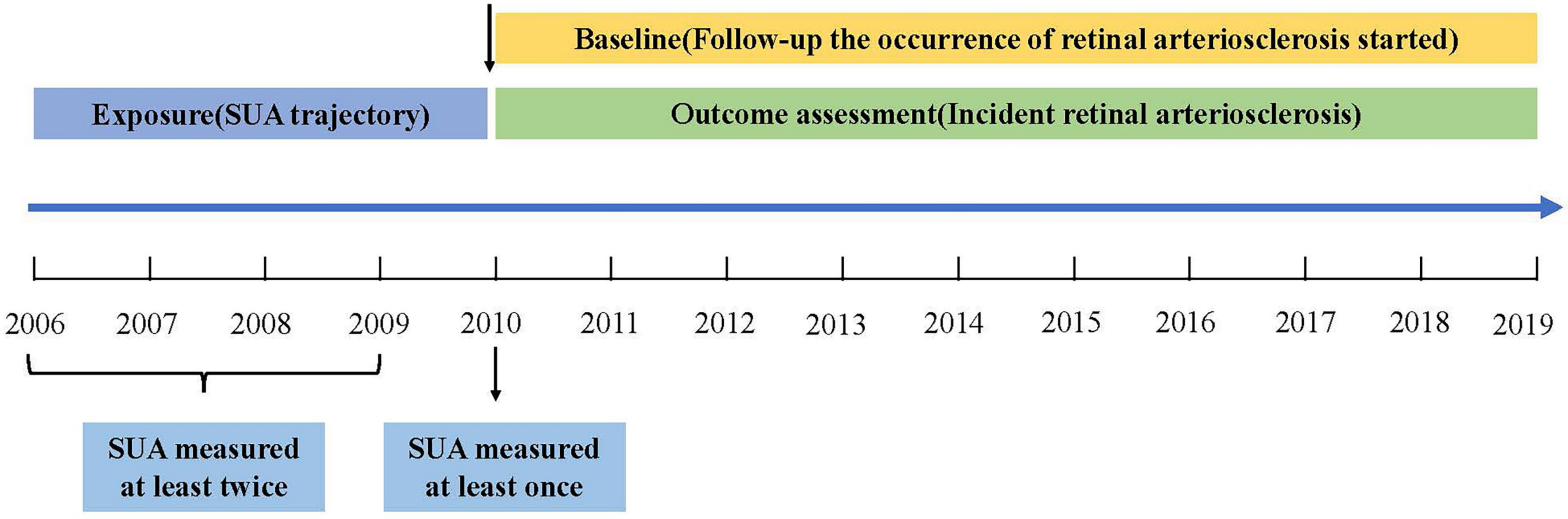
Study design and the time line of exposure and outcome assessment.

**Figure 2:**
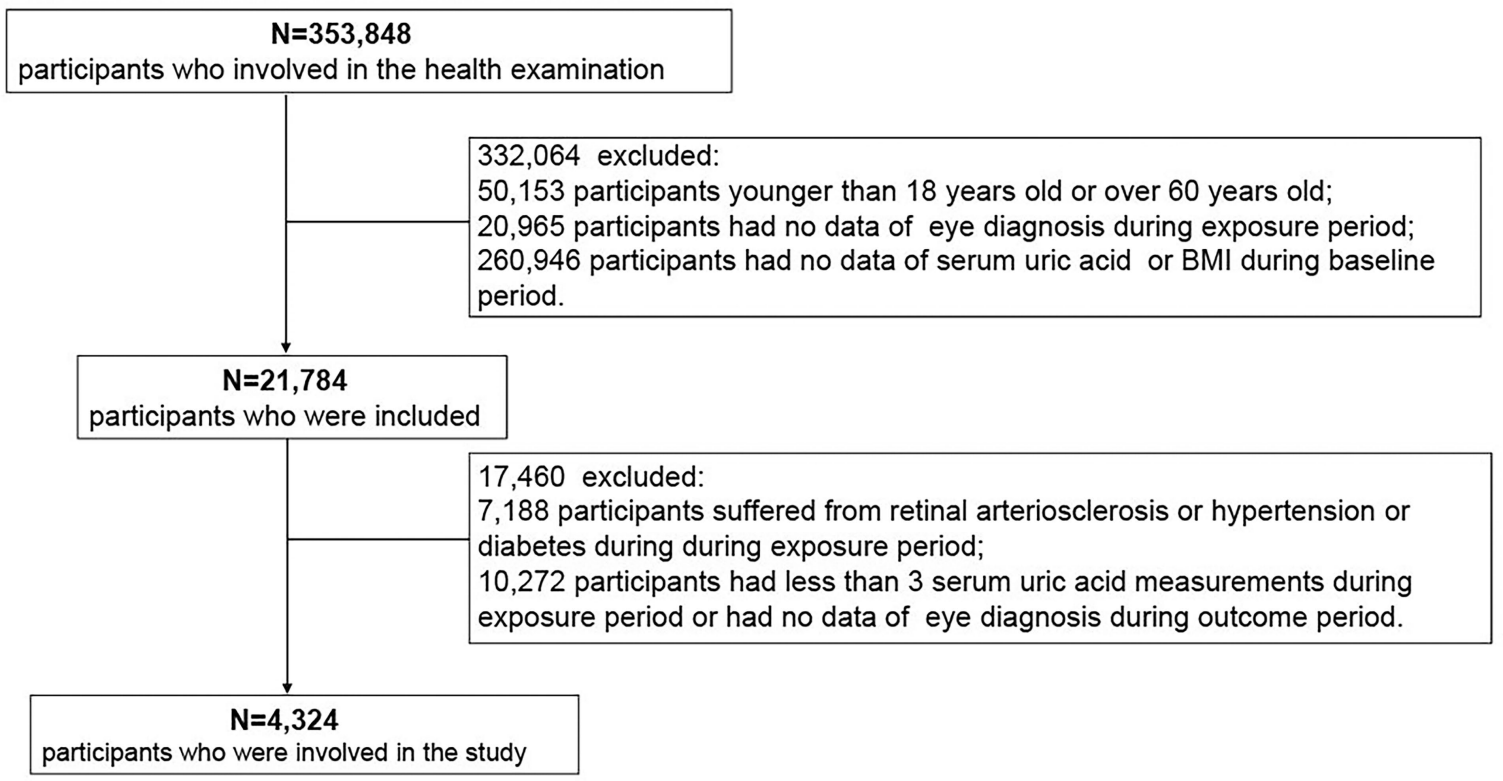
Flow chart of participant inclusion.

This population-based longitudinal study included 353,848 non-manual workers who began undergoing health examinations between 2006 and 2019 at Hua Dong Sanatorium. Most of the subjects were members of organizations or employees of companies. During those years of follow-up, all participants were invited to complete physical examinations, health-related questionnaires, and laboratory tests, and they repeated these tests annually. For this study, 349,524 subjects were excluded based on the following criteria: (i) those who were aged <18 years and ≥ 60 years (considering the diameter of the retinal arteriolar is narrower in the elderly and the degenerative sclerosis in older persons (26)); (ii) those had no information of eye diagnosis during exposure period (from January 1, 2006, through December 31, 2010) (Figure 1) and had no information of SUA or body mass index (BMI) during baseline period (from January 1, 2010, through December 31, 2010); (iii) those suffered from retinal arteriosclerosis or hypertension or diabetes during exposure period (because the previous study reported that high SUA levels significant increase the incident hypertension (10, 11, 27–30), and SUA has been confirmed to play an essential role in the pathogenesis of diabetes (13, 14), what’s more hypertension and diabetes would accelerate the process of retinal arteriosclerosis (2, 3); and (iv) those had less than 3 uric acid measurements during exposure period or had no data of eye diagnosis during outcome period (from January 1, 2011, through December 31, 2019). Thus, a total of 4,324 participants were ultimately included in the analysis of SUA trajectories. Data cleaning steps were presented in Figure 2.

The study was approved by the Ethics Committee and the Institutional Review Board of Hua Dong Sanatorium, Wuxi. All methods were implemented in accordance with the Declaration of Helsinki and the relevant guidelines. The informed consent was waived because the research was a retrospective study, and the need to waive informed consent was also supported by the Ethics Committee of Hua Dong Sanatorium. The personal information of the study subjects was confidential.

### 2.2. Assessment of the SUA

SUA and other biochemical indexes (such as fasting plasma glucose, blood lipids, and creatinine) were determined by AU 5400 BECKMAN COULTER with Enq1zymatic methods at the Hua Dong Sanatorium laboratory.

### 2.3. Assessment of covariates

A demographic characteristics questionnaire, including age, sex, smoking status (current, former, and never), and alcohol drinking status (current, former, and never) was collected by trained staff. The interview also included medical treatment histories, such as hypertension, diabetes, glaucoma, and so on.

Anthropometric measurements were performed by well-trained examiners. The participants were asked to stand up straight, wearing thin, light clothing, and no hats or shoes to measure height and weight. BMI was calculated as weight in kilograms divided by the squared of height in meters. Hypertension was defined as individuals with systolic blood pressure (SBP) ≥ 140 mmHg and/or diastolic blood pressure (DBP) ≥ 90 mmHg or self-reported physician diagnosis of hypertension, or using antihypertensive medications. Diabetes was defined as fasting glucose level ≥ 7.0 mmol/l, or self-reported doctor diagnosed diabetes, or taking oral hypoglycemic drugs or injecting insulin. CVD was defined as definite coronary heart disease, stroke, heart failure, transient ischemic attack, or peripheral arterial disease (12). Blood samples obtained from the anterior cubital vein in the morning after fasting for at least 8 h. The biochemical parameters, including triglycerides (TG), total cholesterol (TC), low-density lipoprotein cholesterol (LDL-C), high-density lipoprotein cholesterol (HDL-C), and creatinine (Cr) were measured by enzymatic methods with an autoanalyzer (AU5400, BECKMAN COULTER). Besides, the estimated glomerular filtration rate (eGFR) was estimated by using the Modification of Diet in Renal Disease (MDRD) equation applied for Chinese patients (28, 31). The equation:

eGFR (mL/min/1.73 m^2^) = 175 × Cr^−1.234^ (mg/ dL) × age^−0.179^(years) × 0.79 (in women).

### 2.4. Assessment of the outcome

The outcome was to assess the sex-specific association of distinct SUA trajectory groups (identified in a general population using SUA measurements between 2006 and 2010) with the subsequent development of retinal arteriosclerosis (Figure 1).

Fundus examinations were performed by trained ophthalmologists. The procedure followed standardized methods (32). Briefly, after 5 min of darkness adaptation, a 45° static retinal photo was obtained with the non-dilated fundus camera TRC.NW400TOPCON. Retinal arteriosclerosis was divided into four grades according to the Keith-Wagener fundus grading method (33). Grade I: spasm or mild sclerosis of the retinal artery; Grade II: the degree of retinal arteriosclerosis was more obvious than that of Grade I. Pathological changes of different degrees could be seen at the arteriovenous junction, and the arterial light reflection was widened, copper and silver filaments were visible; Grade III: in addition to retinal artery stenosis and sclerosis, there was retinal edema, cotton lint spots, rigid exudates, hemorrhagic spots, etc.; Grade IV: in addition to grade III changes, there was also optic disk edema. Grades I ~ IV were all diagnosed as retinal arteriosclerosis (21).

### 2.5. Identification of SUA trajectories

**Figure 3:**
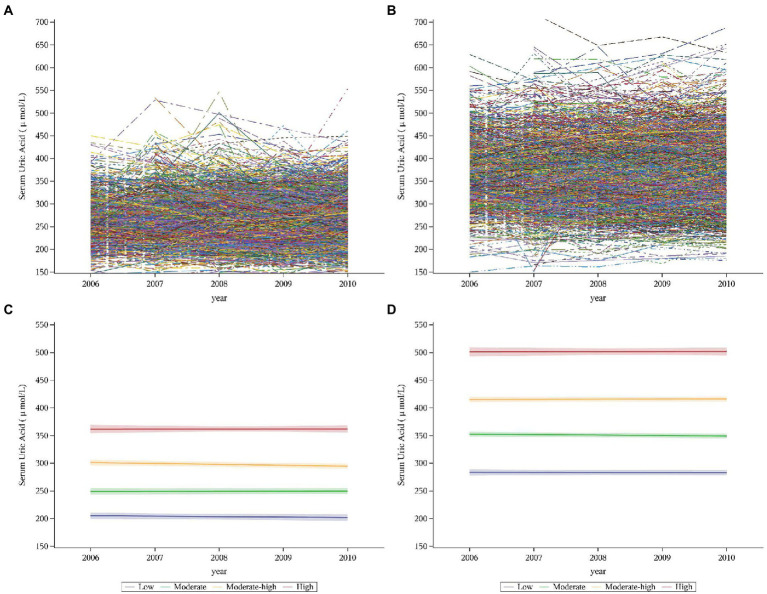
Individual trajectories and fitted trajectories of serum uric acid over 5 years (exposure period). **(A)** Individual trajectories in women. **(B)** Individual trajectories in men. **(C)** Fitted trajectories in women. **(D)** Fitted trajectories in men. Serum uric trajectories in each sex were identified by Group-based Trajectory Modeling (SAS PROC TRAJ), using serum uric acid levels during 2006–2010 exams. Men and women were categorized into 4 trajectories: the low, moderate, moderate-high, and high groups.

Given the substantial differences in SUA levels existing between sexes, we determined SUA trajectories for different sexes. In this study, we identified distinct trajectory groups according to individual SUA trajectories by using group-based trajectory modeling (GBTM). These models were implemented by SAS Proc Traj procedure (34). The GBTM is a specialized application of finite mixture modeling. The aim is to identify clusters of individuals with similar trajectories and thus to determine several subpopulations with different trajectories (35). Several statistically oriented criteria for assessing model fit and the number of trajectories. These include: (i) the higher values of Bayesian Information Criterion (BIC) and Akaike Information Criterion (AIC) (close to zero, indicating a good fit) (36); (ii) the average of the posterior probabilities of each group exceeds 70%; (iii) the number of participants in each trajectory exceeds 5% of the overall population. We intuitively sketched each person’s trajectory, and we could see from the trajectory that each person’s trajectory had undergone various alterations (Figures 3A,B). Then, we constructed models with different numbers and different forms of trajectories (linear, quadratic, or cubic) (37). We started with a model with one trajectory and then, constructed the trajectory models with two, three, four, and five. BIC and AIC increased as the number of trajectory groups increased (Supplementary Figure S1). When the model with five trajectories, the number of participants in several trajectory groups was less than 5%, as the result, four trajectory groups were identified to fit the best. Starting with all these trajectories in cubic and then quadratic and linear, respectively, we then compared the model fit of models with 4 trajectories with different functional forms. In our final model, we had four trajectories with liner order terms, and from this final model, we calculated the greatest posterior predicted probabilities for each individual of being a member of a given trajectory group, and the average probability of final group membership was 0.90 in men (range 0.89–0.92 across the trajectory groups) and 0.87 in women (range 0.84–0.91 across the trajectory groups) (Supplementary Table S1).

### 2.6. Statistical analyzes

Continuous variables were expressed in terms of median (interquartile spacing) and categorical variables in terms of number (percentage). Kolmogorov–Smirnov test was used for the distribution of variables. In order to compare the differences between groups, ANOVA or Kruskal–Wallis test was used for continuous variables, and Chi-square test was used for categorical variables. In accordance with the recommendations for inferences from incomplete data from the National Research Council (38), we assumed that the missing data during follow-up were missing at random, and we performed a Little’s MCAR test. The result showed that the chi-square value was 29.404 and the *p* value was 0.105, which was greater than 0.05. The missing values could be considered to be missing completely at random (MACR). Therefore, the multiple imputation was used to impute the baseline missing values (39). We generated 5 datasets to account for the missing data at baseline (Supplementary Tables S2, S3) and chose the median of these values for final calculations (40). The follow-up time was from the baseline date to the diagnosis date of the retinal arteriosclerosis or the censoring date (December 31, 2019), whichever came first, and the median follow-up time was calculated by reverse Kaplan–Meier method (41). The cumulative incidence curves were estimated by the Kaplan–Meier method, and any differences were evaluated with log-rank tests. To compare the risk of retinal arteriosclerosis in different SUA trajectory groups, univariate and multivariable Cox proportional hazard models were constructed to estimate hazard ratios (HRs) and 95% confidence intervals (CIs) for changes in SUA in women, men, and total populations. The variables included in the multivariate model were statistically significant in the baseline analyzes and were shown to influence outcomes according to previous research. Furthermore, to assess robustness of the observed association between SUA trajectory groups and retinal arteriosclerosis, we examined the effect modification of SUA trajectories for retinal arteriosclerosis development in prespecified subgroups by baseline age (<45 or ≥ 45 years), BMI (<24 or ≥ 24 kg/m^2^) and SBP (<120 or ≥ 120 mmHg).

*P* trend tests were performed by assessing the statistical significance across categorical SUA trajectory groups as an ordinal variable, and to evaluate incidence density trends of retinal arteriosclerosis during the median 9.54-year follow-up period in study population, the Cochran–Armitage test was used (28, 42). Person years were calculated from baseline until the date of diagnosis or the end of follow-up (43).

To evaluate the impact of the continuous changes in SUA on the incidence rate, a multivariable Cox model with restricted cubic splines (RCS) was built. The spline was defined using four knots at the 5th, 25th, 75th, and 95th percentiles (44), and the threshold was determined as the point in SUA level with the smallest hazard ratio (45, 46).

We conducted several sensitivity analyzes. Given the tendency for the trajectories to change smoothly, we further created models to assess serum uric acid levels at a single time point and stratify the baseline SUA according to quartiles instead of the trajectories. We divided participants into four groups based on the quartiles of the cumulative average of SUA levels during the exposure period to investigate the effects of cumulative SUA exposure. Furthermore, we reanalyzed the data after including individuals who had a history of hypertension and diabetes or were over 60 years old during the exposure period, which might affect the outcomes.

All statistical analyzes were performed with SAS version 9.4 (SAS Institute, Cary, NC, United States) and R version 4.1.2 (R Foundation). The two-sided *p* < 0.05 was considered statistically significant.

## 3. Results

### 3.1. The trajectory groups of SUA

A total of 4,324 participants (1,781 women and 2,543 men) were enrolled in our study (Figure 2; Table 1). We identified SUA trajectories by sex-specific: four trajectories in women and four similarly shaped trajectories in men, and as a group, men had higher serum uric acid concentrations than women. We classified trajectories as “low,” “moderate,” “moderate-high,” and “high” based on morphological characteristics. 17.65% of women and 16.35% of men had the low trajectory; 44.89% of women and 45.01% of men had the moderate trajectory; 30.53% of women and 32.19% of men had the moderate-high trajectory; and 6.93% of women and 6.45% of men had the high trajectory (Figures 3C,D; Supplementary Table S1).

**Table 1:** Baseline demographic clinical characteristic according to SUA trajectory groups in women and men.

	SUA trajectory group^*^				
Characteristics	Low	Moderate	Moderate-high	High	*p* Value ^#^
Women					
Total *n* = 1,781					
Age (years)	46.0 (40.0, 50.0)	45.0 (40.0, 51.0)	47.0 (40.0, 52.0)	50.0 (44.0, 54.0)	<0.0001
BMI (kg/m^2^)	21.3 (19.9, 22.8)	21.6 (20.1, 23.2)	22.3 (20.8, 24.3)	23.7 (21.8, 25.8)	<0.0001
SBP (mmHg)	100.0 (100.0, 110.0)	103.3 (100.0, 110.0)	105.0 (100.0, 110.0)	105.0 (100.0, 120.0)	0.0006
DBP (mmHg)	70.0 (60.0, 70.0)	70.0 (60.0, 70.0)	70.0 (64.0, 70.0)	70.0 (65.0, 75.0)	0.0001
Smoking status (%)					
Current	–	2 (0.2)	1 (0.2)	–	>0.9999
Former or never	293 (100.0)	836 (99.8)	528 (99.8)	121 (100.0)	
Alcohol drinking status (%)					
Current	268 (91.5)	750 (89.5)	476 (90.0)	109 (90.1)	0.8166
Former or never	25 (8.5)	88 (10.5)	53 (10.0)	12 (9.9)	
CVD (%)					
Positive	–	5 (0.6)	5 (0.9)	-	0.3829
Negative	293 (100.0)	833 (99.4)	524 (99.1)	121 (100.0)	
Fasting blood concentrations of:					
FBG (mmol/L)	5.1 (4.9, 5.3)	5.1 (4.9, 5.4)	5.1 (4.9, 5.4)	5.3 (5.0, 5.6)	<0.0001
TG (mmol/L)	0.7 (0.6, 1.0)	0.8 (0.6, 1.1)	1.0 (0.7, 1.3)	1.3 (0.9, 1.7)	<0.0001
TC (mmol/L)	4.7 (4.1, 5.3)	4.7 (4.2, 5.2)	4.8 (4.2, 5.4)	5.1 (4.5, 5.6)	<0.0001
LDL-C (mmol/L)	2.8 (2.3, 3.3)	2.8 (2.4, 3.3)	3.0 (2.6, 3.5)	3.3 (2.8, 3.7)	<0.0001
HDL-C (mmol/L)	1.7 (1.5, 1.9)	1.6 (1.4, 1.9)	1.5 (1.3, 1.7)	1.5 (1.2, 1.7)	<0.0001
Cr (μmol/L)	53.2 (49.0, 57.7)	55.3 (50.5, 60.2)	56.0 (51.7, 61.2)	58.5 (54.3, 62.9)	<0.0001
eGFR (mL/min/1.73 m^2^)	167.8 (150.4, 183.9)	158.2 (143.1, 177.8)	155.5 (138.8, 173.3)	146.2 (132.2, 160.8)	<0.0001
SUA (μmol/L)	200.5 (182.2, 215.1)	248.7 (229.3, 265.9)	291.7 (273.3, 316.0)	359.4 (343.1, 384.6)	<0.0001
Men					
Total *n* = 2,543					
Age (years)	48.0 (42.0, 53.0)	46.0 (40.0, 51.0)	46.0 (41.0, 51.0)	46.0 (40.0, 51.0)	0.0065
BMI (kg/m^2^)	22.9 (21.1, 24.7)	24.0 (22.2, 25.7)	25.1 (23.5, 26.6)	25.4 (23.9, 27.1)	<0.0001
SBP (mmHg)	110.0 (105.0, 120.0)	110.0 (110.0, 120.0)	115.0 (110.0, 120.0)	115.0 (110.0, 120.0)	<0.0001
DBP (mmHg)	70.0 (68.0, 76.0)	70.0 (70.0, 80.0)	70.0 (70.0, 80.0)	75.0 (70.0, 80.0)	<0.0001
Smoking status (%)					
Current	187 (45.8)	536 (46.1)	397 (48.8)	72 (45.3)	0.5979
Former or never	221 (54.2)	627 (53.9)	416 (51.2)	87 (54.7)	
Alcohol drinking status (%)					
Current	131 (32.1)	292 (25.1)	149 (18.3)	30 (18.9)	<0.0001
Former or never	277 (67.9)	871 (74.9)	664 (81.7)	129 (81.1)	
CVD (%)					
Positive	1 (0.2)	10 (0.9)	6 (0.7)	2 (1.3)	0.4654
Negative	407 (99.8)	1,153 (99.1)	807 (99.3)	157 (98.7)	
Fasting blood concentrations of:					
FBG (mmol/L)	5.2 (5.0, 5.5)	5.3 (5.0, 5.5)	5.3 (5.1, 5.6)	5.4 (5.1, 5.8)	<0.0001
TG (mmol/L)	1.2 (0.8, 1.6)	1.3 (1.0, 1.8)	1.7 (1.2, 2.4)	2.1 (1.5, 3.0)	<0.0001
TC (mmol/L)	4.8 (4.2, 5.3)	4.8 (4.2, 5.3)	5.0 (4.5, 5.5)	5.0 (4.5, 5.6)	<0.0001
LDL-C (mmol/L)	3.1 (2.6, 3.7)	3.2 (2.7, 3.6)	3.3 (2.8, 3.8)	3.4 (2.8, 3.9)	<0.0001
HDL-C (mmol/L)	1.3 (1.1, 1.6)	1.3 (1.1, 1.5)	1.2 (1.0, 1.4)	1.1 (1.0, 1.3)	<0.0001
Cr (μmol/L)	74.2 (68.7, 80.8)	75.6 (69.7, 81.7)	77.1 (71.1, 83.3)	81.2 (73.8, 87.8)	<0.0001
eGFR (mL/min/1.73 m^2^)	86.4 (77.4, 95.9)	84.8 (77.2, 94.2)	82.5 (75.0, 91.6)	78.2 (70.6, 89.0)	<0.0001
SUA (μmol/L)	284.9 (260.3, 302.7)	347.7 (326.1, 369.1)	415.6 (392.4, 440.4)	495.9 (475.6, 526.7)	<0.0001

Continuous variables are expressed as median (interquartile range). Categorical variables are expressed as frequency (percent).

* SUA trajectory groups were determined by a group-based trajectory modeling using SUA readings during the exposure period, among which the model with the highest discrimination ability between trajectory groups was selected. The trajectory groups were named as “low, moderate, moderate-high and high” according to visual appearance of SUA trends.

# For continuous variables with normal distribution using one-way ANOVA, Kruskal–Wallis test for non-normally distributed variables, chi-square test for categorical variables.

BMI, body mass index; SBP, systolic blood pressure; DBP, diastolic blood pressure; CVD, cardiovascular disease; FBG, fasting blood glucose; TG, triglycerides; TC, total cholesterol; LDL-C, low density lipoprotein; HDL-C, high density lipoprotein; Cr, creatinine; eGFR, estimated glomerular filtration rate; SUA, serum uric acid.

### 3.2. Patient characteristics of various SUA trajectories at baseline

Table 1 shows the baseline characteristics stratified by SUA trajectories. Among both women and men, the high trajectory group had a higher BMI. In addition, there was a statistical difference in age, BMI, SBP, DBP, FBG, TG, TC, LDL-C, HDL-C, Cr, and eGFR among SUA trajectory groups. However, there was negatively related to smoking status and alcohol drinking status in women, and positively related to alcohol drinking status in men.

### 3.3. Incidence of retinal arteriosclerosis

Table 2 presents the incidence densities of retinal arteriosclerosis development according to SUA trajectory groups in women and men. We observed that 97 women developed retinal arteriosclerosis during a median follow-up of 9.54 years (IQR 9.53–9.56), and 295 men developed retinal arteriosclerosis, with an overall incidence rate of 8.8 per 1,000 person-years in women. There were 13 (4.4%), 38 (4.5%), 33 (6.2%), and 13 (10.7%) retinal arteriosclerosis events in the low, moderate, moderate-high, and high SUA trajectory groups, respectively, with corresponding incidence densities of 6.8 (95% CI, 3.1–10.5), 7.3 (95% CI, 5.0–9.6), 10.3 (95% CI, 6.8–13.8), and 19.7 (95% CI, 9.1–30.3) per 1,000 person-years, respectively. And the Cochran-Armitage trend test showed that retinal arteriosclerosis incidence was significantly elevated as SUA changing trajectory increased (*p* for trend = 0.0104). Analogously, in men, the overall incidence was 17.9 per 1,000 person-years, and there were 31 (7.6%), 115 (9.9%), 125 (15.4%), and 24 (15.1%) retinal arteriosclerosis events in the low, moderate, moderate-high, and high SUA trajectory groups, respectively, with corresponding incidence densities of 11.9 (95% CI, 7.7–16.0), 15.4 (95% CI, 12.6–18.2), 23.4 (95% CI, 19.3–27.4), and 23.2 (95% CI, 14.0–32.3) per 1,000 person-years, respectively. The incidence of retinal arteriosclerosis trend was similar in women (*p* for trend < 0.001). However, in each corresponding trajectory group, the incidence of retinal arteriosclerosis was generally higher in men than in women.

**Table 2:** Incidence densities of retinal arteriosclerosis development according to SUA trajectory groups in women and men.

		SUA trajectory group				
Variables	All	Low	Moderate	Moderate-high	High	*p* for trend^*^
Women						
Participants, *n*	1,781	293	838	529	121	
Participants, *n* (person-years)	11010.5	1901.5	5239.3	3210.6	659.1	
Incidence, *n*	97	13	38	33	13	
Incidence density per 1,000 person-years (95% CI)	8.8 (7.1,10.6)	6.8 (3.1,10.5)	7.3 (5.0,9.6)	10.3 (6.8,13.8)	19.7 (9.1,30.3)	0.0104
Men						
Participants, n	2,543	408	1,163	813	159	
Participants, n (person-years)	16459.9	2608.8	7470.5	5344.1	1036.6	
Incidence, n	295	31	115	125	24	
Incidence density per 1,000 person-years (95% CI)	17.9 (15.9,19.9)	11.9 (7.7,16.0)	15.4 (12.6,18.2)	23.4 (19.3,27.4)	23.2 (14.0,32.3)	<0.0001

* Tests of trend were conducted by the Cochran–Armitage test.

**Figure 4:**
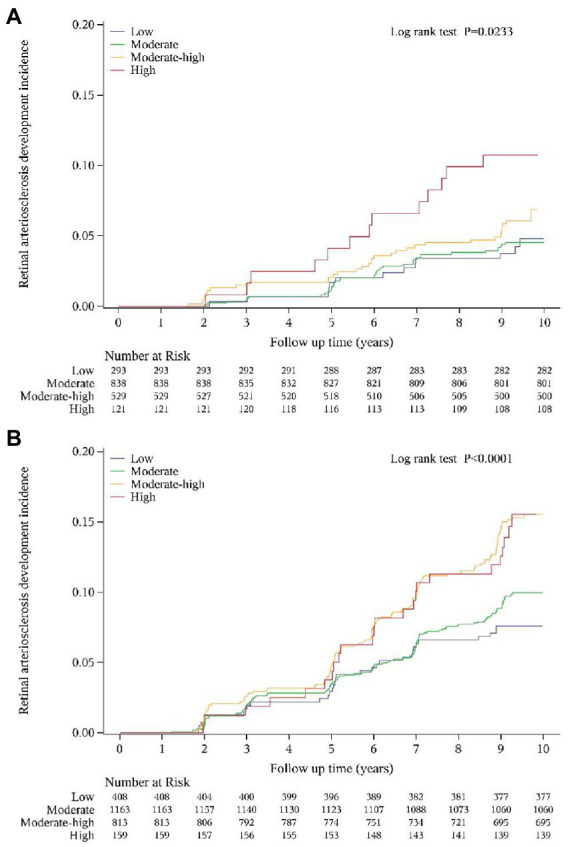
Cumulative incidence curve for retinal arteriosclerosis according to SUA trajectory groups in women **(A)** and men **(B)**.

The cumulative incidence curves also showed that the retinal arteriosclerosis event-free survival rate was significantly lower in the high trajectory group and moderate-high trajectory group than in the other two groups (log-rank test: *p* = 0.0023 in women; *p* < 0.001 in men) (Figure 4).

### 3.4. Association of SUA trajectory groups and retinal arteriosclerosis risk

Cox proportional-hazards models were conducted to evaluate the relationship between SUA trajectory groups and retinal arteriosclerosis risk (Table 3). In the unadjusted Cox proportional-hazards models, higher SUA trajectory groups were associated with new-onset retinal arteriosclerosis compared with those in the low SUA trajectory group in women and men. All associations were attenuated after adjustment for age, BMI, and other covariates. After adjusting for potential confounders, these associations remained significant only in men. We found that, in men, the relationship between moderate-high and high SUA trajectory groups and retinal arteriosclerosis was statistically significant in all models. And the high trajectory group experienced a greater hazard ratio than the moderate-high trajectory group. Besides, the retinal arteriosclerosis risk increases with the SUA changing trajectory increases in those models (*p* for trend<0.05). The association of SUA trajectory groups with retinal arteriosclerosis differed in men and women. Overall, the HRs with 95% CI for moderate, moderate-high trajectory group, and high trajectory group were 1.14 (95% CI, 0.81–1.60), 1.42 (95% CI, 1.00–2.01), and 1.38 (95% CI, 0.87–2.20) compared with the low trajectory group, respectively. We also constructed uric acid trajectories in the total population separately, and the results of the trajectories are shown in Supplementary Figure S2. The relationship between SUA and retinal arteriosclerosis in the total population was also not statistically significant (Supplementary Table S4).

**Table 3:** Hazard ratios of retinal arteriosclerosis development according to SUA trajectory groups in sex-specific study population.

	Model 1		Model 2	
Variables	HR, (95%CI)	*p* Value	HR, (95%CI)	*p* Value
Women				
Low	1.00 (ref)	–	1.00 (ref)	–
Moderate	1.03 (0.55,1.93)	0.9351	0.86 (0.46,1.64)	0.6563
Moderate-high	1.43 (0.75,2.72)	0.2734	0.77 (0.39,1.52)	0.4587
High	2.53 (1.17,5.45)	0.0182	0.73 (0.31,1.75)	0.4832
*p* for trend^*^		0.0095		0.4263
Men				
Low	1.00 (ref)	–	1.00 (ref)	–
Moderate	1.31 (0.88,1.94)	0.1853	1.29 (0.86,1.93)	0.2139
Moderate-high	2.09 (1.41,3.10)	0.0002	1.76 (1.17,2.65)	0.0068
High	2.04 (1.20,3.48)	0.0086	1.81 (1.04,3.17)	0.0367
*p* for trend^*^		<0.0001		0.0021
Pooled				
Low	1.00 (ref)	–	1.00 (ref)	–
Moderate	1.22 (0.87,1.71)	0.2391	1.14 (0.81,1.60)	0.4481
Moderate-high	1.93 (1.38,2.69)	0.0001	1.42 (1.00,2.01)	0.0492
High	2.18 (1.41,3.37)	0.0005	1.38 (0.87,2.20)	0.1681
*p* for trend^*^		<0.0001		0.0318

Model 1: Not adjust.

Model 2: Adjustment for age, body mass index, sex, systolic blood pressure, diastolic blood pressure, alcohol drinking status (in men and total-people), triglyceride level, total cholesterol, low-density lipoprotein cholesterol level, high-density lipoprotein cholesterol level, creatinine, and estimated glomerular filtration rate at baseline (from June 1, 2010, through June 1, 2011).

No adjustment for sex in men and women.

ref: reference.

* Tests of trend were conducted by assessing the statistical significance across categorical SUA trajectory groups as an ordinal variable.

**Figure 5:**
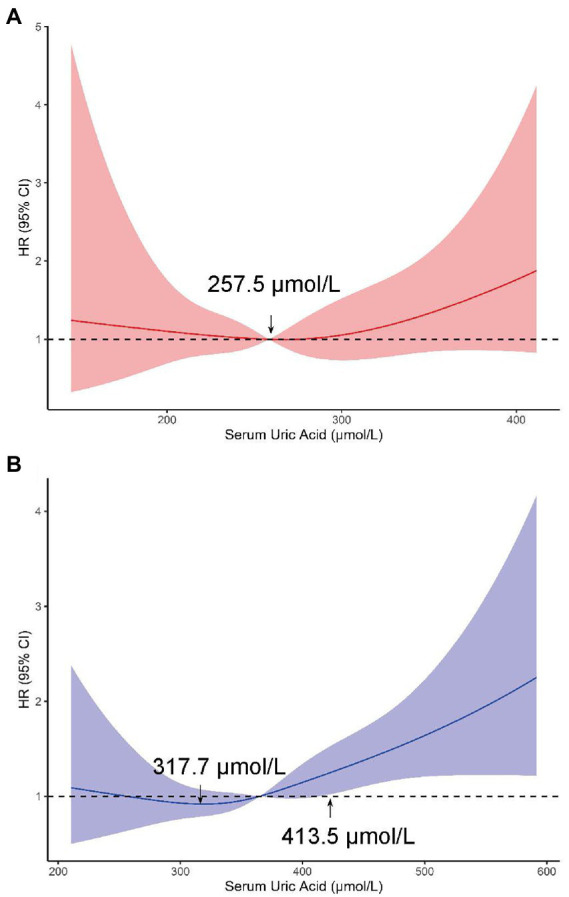
Adjusted HRs (95% CIs) for retinal arteriosclerosis incidence from restricted cubic splines in women **(A)** and **(B)** men. These models were adjusted for age, body mass index, systolic blood pressure, diastolic blood pressure at baseline (from June 1, 2010, through June 1, 2011), alcohol drinking status (in men), triglyceride level, total cholesterol, low-density lipoprotein cholesterol level, high-density lipoprotein cholesterol level, creatinine and estimated glomerular filtration rate.

Spline regression analyzes also showed a graded association of changes in SUA with the risk of incident retinal arteriosclerosis. Similar to the results of the above model, increasing SUA was associated with a higher risk of retinal arteriosclerosis development in men. The plot showed a slow reduction of the risk within the lower range of SUA, which reached the lowest risk around 317.7 μmol/l and then increased thereafter. The concentration of SUA associated with the lowest risk of retinal arteriosclerosis in multivariable adjusted analyzes was 413.5 μmol/l in the men. However, this did not reach statistical significance in women (Figure 5).

We further analyzed the association of SUA trajectory groups with retinal arteriosclerosis development. We examined the effect modification by an *a priori*–selected set of baseline characteristics, including baseline age (<45 or ≥45 years), SBP (<120 or ≥120 mmHg), BMI (< 24 or ≥ 24 kg/m^2^). The number of patients and incidence density in different subgroups are shown in Supplementary Table S5. *p* values for interaction were not significant for all subgroups except the age subgroup. The significant association of SUA trends with the retinal arteriosclerosis outcome was consistent across subgroups by BMI and SBP. Which was higher SUA trajectory groups were associated with new-onset retinal arteriosclerosis in men but not in women. In men, in the age subgroup with, test for interaction showed that the effect of SUA level on retinal arteriosclerosis was significantly between age subgroup (< 45 or ≥ 45 years) (*p* value for interaction = 0.0031). The effect of SUA on outcome was greater at ages older than 45 years. In the subgroup with BMI ≥ 24 kg/m^2^, participants with moderate, moderate-high or high SUA trajectory had a 2.05-fold, 2.58-fold, and 2.52-fold higher risk of retinal arteriosclerosis than those with a low trajectory. In the subgroup with SBP ≥ 120 mmHg, moderate-high trajectory group and high trajectory group were associated with a high risk of retinal arteriosclerosis development. In contrast, in the subgroup with age < 45 years, BMI < 24 kg/m^2^, and SBP < 120 mmHg, there was no statistically significant relationship between SUA trajectory groups and retinal arteriosclerosis. However, in women, higher SUA trajectory groups were relatively protective compared to the low group, which may be due to chance and needs further exploration (Supplementary Figures S3, S4).

### 3.5. Sensitivity analysis

To substantiate the robust association of SUA trajectory groups with the subsequent outcome, several sensitivity analyzes were conducted in this study. Results of all sensitivity analyzes were largely consistent. In men, we observed that high or moderate-high SUA trajectory groups were significantly associated with future retinal arteriosclerosis risk. Whereas, there was no association in women. First, in the analyzes using quartiles at baseline level instead of SUA trajectories, we found a similar association between high SUA levels with risk of retinal arteriosclerosis development in men, but not in women. Second, when the cumulative average of SUA levels during the exposure period was divided into four groups, the associations of SUA levels with retinal arteriosclerosis risk remained significant in men. Finally, we additionally performed analyzes using the complete dataset, including people over 60 years of age and people with hypertension or diabetes, and found the similar results, high SUA trajectory group was associated with a 1.43-fold higher risk of retinal arteriosclerosis development in men (Table 4).

**Table 4:** Adjusted HRs (95% CIs) of retinal arteriosclerosis development in sensitivity analyses.

	Low	Moderate	Moderate-high	High
Women				
Sensitivity analysis 1^*^	1.00 (ref)	0.84 (0.44,1.61)	0.71 (0.37,1.36)	1.08 (0.59,1.98)
Sensitivity analysis 2^#^	1.00 (ref)	1.32 (0.71,2.45)	0.65 (0.32,1.33)	1.46 (0.78,2.72)
Sensitivity analysis 3	1.00 (ref)	0.97 (0.66,1.43)	1.00 (0.66,1.51)	1.48 (0.89,2.45)
Men				
Sensitivity analysis 1^*^	1.00 (ref)	0.87 (0.60,1.25)	1.09 (0.77,1.54)	1.44 (1.02,2.03)
Sensitivity analysis 2^#^	1.00 (ref)	0.97 (0.66,1.42)	1.41 (0.99,2.00)	1.69 (1.19,2.41)
Sensitivity analysis 3	1.00 (ref)	1.12 (0.90,1.38)	1.33 (1.07,1.66)	1.43 (1.08,1.90)

Model: adjusted for age, body mass index, systolic blood pressure, diastolic blood pressure, alcohol drinking status (in men), triglyceride level, total cholesterol, low-density lipoprotein cholesterol level, high-density lipoprotein cholesterol level, creatinine, and estimated glomerular filtration rate at baseline (from June 1, 2010, through June 1, 2011).

Sensitivity analysis 1, the baseline SUA levels instead of the SUA trajectories.

Sensitivity analysis 2, the average SUA levels during the exposure period instead of the SUA trajectories.

Sensitivity analysis 3, people with hypertension or diabetes and older than 60 during the exposure period were included (further adjustment for hypertension and diabetes).

* The baseline SUA levels were divided to four groups according to the quartiles, Q1 corresponds to Low, Q2 corresponds to Moderate, Q3 corresponds to Moderate-high, Q4 corresponds to High. Serum uric acid quartiles (Q1–Q4) in women (Q1: < 227.20; Q2: 227.20–258.40; Q3: 258.40–289.70; and Q4: > 435.20 μ mol/L). In men (Q1: < 322.30; Q2: 322.30–365.00; Q3: 365.00–411.90; and Q4: > 411.90 μ mol/L).

# The average SUA levels during the exposure period were divided to four groups according to the quartiles, Q1 corresponds to Low, Q2 corresponds to Moderate, Q3 corresponds to Moderate-high, Q4 corresponds to High. Serum uric acid quartiles (Q1–Q4) in women (Q1: < 231.95; Q2: 231.95–260.60; Q3: 260.60–292.33; and Q4: > 292.33 μ mol/L). In men (Q1: < 328.30; Q2: 328.30–368.02; Q3: 368.02–407.97; and Q4: > 407.97 μ mol/L).

## 4. Discussion

In this population-based longitudinal study of Chinese adults without diabetes and hypertension, we identified 4 SUA trajectory groups in women and men during a 5-year exposure period: low, moderate, moderate-high, and high. Besides, in the multivariate model, higher trajectory groups were positively associated with retinal arteriosclerosis risk, but only in men. In addition, spline regression analyzes also suggested that this relationship was more robust among participants with higher SUA levels. In men, these observed associations between SUA trajectory groups and retinal arteriosclerosis were independent of BMI, age, blood pressure, and lipid parameters. However, in women, the association of higher SUA trajectory groups with retinal arteriosclerosis events was attenuated to non-significance after adjustment for covariates.

To our knowledge, this is the first study to date to assess the associations between SUA trajectory groups and the risk of retinal arteriosclerosis. Although there have been many studies on the prevalence of retinal arteriosclerosis (1, 21, 47), there are fewer studies reporting on the incidence. Our study found that the incidence of retinal arteriosclerosis in Chinese men was higher than women. The incidence densities of retinal arteriosclerosis were 17.9 per 1,000 person-year (95% CI: 15.9–19.9) for men and 8.8 (95% CI: 7.1–10.6) for women. Retinal arteriosclerosis is a microvascular disease of arteriosclerosis, in order to exclude the influence of traditional factors on arterial stiffness, hypertension and diabetes were excluded in this study. And our study showed that the association between SUA trajectory groups and retinal arteriosclerosis was statistically significant only in men. SUA has been linked to retinal arteriosclerosis in previous cross-sectional analyzes (21). The cross-sectional nature of prior investigations limits the ability to understand the temporal association of serum uric acid with retinal arteriosclerosis. Our findings demonstrated that SUA in early exposure period, and its patterns of change over time were associated with retinal arteriosclerosis. Besides, the sex discrepancy seen in the cross-sectional research has been borne out in our study and supported that SUA plays a greater role in the pathogenesis of retinal arteriosclerosis in men than in women. The sex difference has also been reported in previous studies. Another cross-sectional population-based study of 779 subjects showed that the high-normal SUA levels were associated with an increased risk of arterial stiffness in healthy Korean men, with an OR of 2.91 (95% CI: 1.39–6.11) for 4th vs. 1st SUA quartiles (20). A prospective research including 3,686 subjects found a significant positive correlation between SUA levels and cIMT, a recognized marker of atherosclerosis, in men (48). Whereas, a cross-sectional study conducted by Chen et al. (including 12,988 subjects) found that hyperuricemia was related to a higher risk of atherosclerotic cardiovascular disease in both sexes, and the relationship was much stronger in women than in men. This discrepancy may be due to Chen’s study that almost all women with high levels of uric acid were over 45 years old and likely to be in menopause. As the secretion of estrogen decreases with age and its protective effect on the cardiovascular system may gradually diminish, making it more susceptible to elevated uric acid levels (19). We observed that the HRs for retinal arteriosclerosis in BMI ≥ 24Kg/m^2^, moderate-high trajectory group and high trajectory group were 2.58,2.52, respectively. However, there was no statistically significant relationship between high SUA levels and retinal arteriosclerosis when BMI < 24Kg/m^2^. Indeed, the previous study has reported that overweightness or obesity appeared significantly associated with increased arterial stiffness (49). In addition, subgroup analyzes also showed that positive association between SUA trajectory groups and retinal arteriosclerosis development was observed only in SBP ≥ 120 mmHg. Hypertension is a well-known risk factor for retinal arteriosclerosis (3), and it has been reported that blood pressure indexes had high predictive performance for arteriosclerosis in eastern Chinese adults, and the optimal cut-off point for SBP was 123.5 mmHg (50). Further studies are needed to confirm the specific SBP cut-off point in other populations and settings.

The mechanisms by which SUA reflects the risk for retinal arteriosclerosis are not fully understood, even though previous studies have been done in this area. High uric acid levels might generate superoxide and oxidative stress *via* the xanthine oxidase pathway and promote the development of atherosclerosis by stimulating inflammation (20). In addition, experimental evidence suggested that adverse effects of SUA on the vasculature were associated with increased chemokine and cytokine expressions, induction of the renin-angiotensin system, and increased vascular C-reactive protein (CRP) expression (51). SUA also reduced nitric oxide (NO) bioavailability, ensuring endothelial dysfunction, inflammation, and vasoconstriction (52, 53). Vitro and *in vivo* data also suggested that hyperuricemia disturbs the balance of the asymmetric dimethylarginine / dimethylarginine dimethylaminotransferases-2 axis, results in endothelial cell dysfunction, and, consequently, accelerates atherosclerosis (54). One animal experiment has shown that high SUA levels promote atherosclerosis by targeting NRF2-mediated autophagy dysfunction and ferroptosis (55). A pathophysiological hypothesis may explain these sex differences, SUA metabolism is genetically regulated, and sex differences play an important role in regulating SUA concentrations. Distinct patterns of metabolic alteration between sexes, possibly due to different sex hormones exposure, may influence men to be more susceptible the deleterious effects of SUA levels on atherosclerosis risk (48). However, it has also been shown that SUA was considered an antioxidant and could be expected to benefit the cardiovascular system (56). Therefore, it is necessary to conduct a large cohort study to investigate the role of SUA levels in the progression of atherosclerosis.

Sensitivity analyzes revealed the association between SUA trajectory groups and retinal arteriosclerosis incidence was not affected by age and the presence of hypertension or diabetes. These were included in the sensitivity analyzes, and the results were consistent with the overall results. In addition, the clinical reference value of SUA is 420 μmol/l in men and 360 μmol/l in women (25), the sensitivity analyzes of the entire study demonstrated that SUA around or greater than 412 μmol/l was a risk factor for retinal arteriosclerosis development in men. Although the trajectories changed smoothly, if a single time point was used to predict, the median of SUA to achieve statistical significance was around 440 μmol/l and not accurate enough (Table 5). Thus, SUA trajectories did predict incident retinal arteriosclerosis better than serum uric acid at a single time point. We also did observe differences in the patterns of risk in men and women. Nevertheless, it must be considered that SUA levels in women in this study were almost below the clinical reference, the diagnostic criteria for hyperuricemia, therefore we cannot rule out an association between SUA levels and retinal arteriosclerosis progression in women, which would require longer follow-up.

**Table 5:** Serum uric acid levels at each sensitivity analysis of SUA (**μ**mol/L) concentration.

	Serum uric acid, median (interquartile range), μmol/L			
	Low	Moderate	Moderate-high	High
Women				
Main analysis	200.5 (182.2, 215.1)	248.7 (229.3, 265.9)	291.7 (273.3, 316.0)	359.4 (343.1, 384.6)
Sensitivity analysis 1^*^	206.3 (191.2, 218.3)	243.6 (235.9, 251.5)	272.7 (265.4, 280.4)	320.7 (302.7, 348.9)
Sensitivity analysis 2 ^#^	213.0 (199.0, 223.5)	246.3 (239.9, 253.4)	274.5 (267.6, 283.2)	316.2 (302.4, 339.3)
Sensitivity analysis 3	205.8 (187.9, 223.2)	257.2 (238.2, 275.5)	309.2 (285.5, 332.6)	382.3 (359.9, 411.6)
Men				
Main analysis	284.9 (260.3, 302.7)	347.7 (326.1, 369.1)	415.6 (392.4, 440.4)	495.9 (475.6, 526.7)
Sensitivity analysis 1^*^	296.0 (271.7, 310.2)	343.5 (333.8, 354.6)	386.3 (375.6, 397.8)	447.1 (426.6, 476.7)
Sensitivity analysis 2^#^	302.6 (280.8, 316.8)	349.4 (338.6, 358.5)	386.4 (377.5, 396.8)	439.8 (422.7, 466.7)
Sensitivity analysis 3	283.5 (261.0, 302.2)	348.5 (327.4, 371.5)	412.6 (389.9, 437.9)	486.1 (463.8, 513.5)

Sensitivity analysis 1, the baseline SUA levels instead of the SUA trajectories.

Sensitivity analysis 2, the average SUA levels during the exposure period instead of the SUA trajectories.

Sensitivity analysis 3, people with hypertension or diabetes and older than 60 during the exposure period were included (further adjustment for hypertension and diabetes).

* The baseline SUA levels were divided to four groups according to the quartiles, Q1 corresponds to Low, Q2 corresponds to Moderate, Q3 corresponds to Moderate-high, Q4 corresponds to High. Serum uric acid quartiles (Q1–Q4) in women (Q1: < 227.20; Q2: 227.20–258.40; Q3: 258.40–289.70; and Q4: > 435.20 μ mol/L). In men (Q1: < 322.30; Q2: 322.30–365.00; Q3: 365.00–411.90; and Q4: > 411.90 μ mol/L).

# The average SUA levels during the exposure period were divided to four groups according to the quartiles, Q1 corresponds to Low, Q2 corresponds to Moderate, Q3 corresponds to Moderate-high, Q4 corresponds to High. Serum uric acid quartiles (Q1–Q4) in women (Q1: < 231.95; Q2: 231.95–260.60; Q3: 260.60–292.33; and Q4: > 292.33 μ mol/L). In men (Q1: < 328.30; Q2: 328.30–368.02; Q3: 368.02–407.97; and Q4: > 407.97 μ mol/L).

The study also has clinical implications. As described above, previous studies evaluating the relationship between retinal arteriosclerosis and CVD have demonstrated that retinal arteriosclerosis is associated with a high incidence of CVD, including micro-vascular diseases (2–9). Studies have shown retinal microvasculature may play a role in the pathogenesis of atherosclerosis (57), and retinal arteriosclerosis can also be considered as a marker of systemic vascular aging (1). Thus, from a public health perspective, it is clinically important to stratify the risk of developing retinal arteriosclerosis and identify the high-risk group. However, considering the inconvenience of retinal examination or the disadvantages of computed tomography in assessing CVD, such as radiation exposure and cost, it was not practical to use computed tomography as a tool for risk assessment in the general population. In this study, we found that assessment of SUA levels might help identify individuals at high risk for future retinal arteriosclerosis and subsequent CVD development. SUA examinations can be performed on the general population because this examination is inexpensive and minimally invasive. Therefore, SUA examinations may hold the most promise as a simple assessment tool for evaluating potential atherosclerosis and subsequent risk stratification for CVD risk, even in a busy clinical setting and in the general population.

The strengths and limitations of this study are worth noting. Major strengths of this study are the large sample size and long follow-up of cohorts among the Chinese. The large sample size also allowed us to perform the sex-stratified analyzes with sufficient statistical power. In addition, we constructed the method of GBTM to evaluate the relations of SUA and incident retinal arteriosclerosis. We also conducted a series of sensitivity analyzes to show the robustness of the findings. Nevertheless, we also acknowledge several limitations. First, information on covariates was only used at baseline, we did not capture the long-term covariates trajectories changes. Future studies with repeated measurements are preferred. Second, SUA levels were affected by diet changes and drugs (58, 59). For example, diuretic could affect the SUA levels and was protective factors in fatal myocardial infarction (60). There was also a study showed diuretic-related hyperuricemia was not associated with a higher risk of CVD (59). However, we did not collect dietary information and specific information on SUA-lowering drugs and could not assess the changes of SUA levels were intentional or unintentional in the current study, which limited our ability to further investigate the associations between SUA and retinal arteriosclerosis. And a variety of potential confounding factors should be accounted in the future study. Third, the examination date was used to define the incident date instead of the exact event date. Lastly, given the irregular physical examination of the participants, most of them were excluded from the study, and this study was based on cohorts from a single hospital. Which limits our ability to assess the association between SUA trajectories and retinal arteriosclerosis. In view of this, the generalization of the association needs to be verified by multi-center and larger population studies. Despite these limitations, our results provide important insights into the incidence of retinal arteriosclerosis and its relationship with SUA trajectories and have clinical importance for retinal arteriosclerosis prevention in reminding men to pay attention to SUA levels and its changing trajectory.

In conclusion, our study identified 4 distinct trajectories of SUA in women and men and the findings indicate that SUA trajectory groups differ in their associations with retinal arteriosclerosis. Higher SUA trajectory groups were found to correlate with incident retinal arteriosclerosis in men, whereas such association did not reach statistical significance in women, suggesting that these high-risk persons should be pour closer attention to SUA management to prevent retinal arteriosclerosis.

## Data availability statement

The raw data supporting the conclusions of this article will be made available by the authors, without undue reservation.

## Ethics statement

The studies involving human participants were reviewed and approved by Ethics Committee of Hua Dong Sanatorium. Written informed consent for participation was not required for this study in accordance with the national legislation and the institutional requirements.

## Author contributions

RG, YH, and ZT contributed to the conceptualization and project administration. QF, MJ, and YH collected participant information. RG and ZT analyzed the data. RG and QF wrote the manuscript, with assistance from MJ, YD, SX, CL, YH, and ZT. All authors contributed to the article and approved the submitted version.

## Funding

This study was supported by the National Natural Science Foundation of China (81773541), funded from the Priority Academic Program Development of Jiangsu Higher Education Institutions at Soochow University, the State Key Laboratory of Radiation Medicine and Protection (GZK1201919) to ZT. The Clinical Special Program of Shanghai Municipal Health Commission (Grant number: 20194Y0437), China.

## Conflict of interest

The authors declare that the research was conducted in the absence of any commercial or financial relationships that could be construed as a potential conflict of interest.

## Publisher’s note

All claims expressed in this article are solely those of the authors and do not necessarily represent those of their affiliated organizations, or those of the publisher, the editors and the reviewers. Any product that may be evaluated in this article, or claim that may be made by its manufacturer, is not guaranteed or endorsed by the publisher.

## Abbreviations

BMI: Body mass index
CI: Confidence interval
CIs: Confidence intervals
Cr: Creatinine
CRP: C-reactive protein
CVD: Cardiovascular disease
CVDs: Cardiovascular diseases
DBP: Diastolic blood pressure
eGFR: estimated glomerular filtration rate
FBG: Fasting blood glucose
GBTM: Group-based trajectory modeling
HDL-C: High-density lipoprotein
HR: Hazard ratio
HRs: Hazard ratios
LDL-C: Low-density lipoprotein
MACR: Missing completely at random
MDRD: Modification of Diet in Renal Disease
NO: Nitric oxide
SBP: Systolic blood pressure
SUA: Serum uric acid
TC: Total cholesterol
TG: Triglycerides
