# Supplementary material for: Sex-specific association of serum uric acid trajectories with risk of incident retinal arteriosclerosis in Chinese population: A population-based longitudinal study
Source: Front Cardiovasc Med. 2023 Feb 28;10:1116486. doi: 10.3389/fcvm.2023.1116486 (PMC10011080; doi:10.3389/fcvm.2023.1116486)
Supplement: Supplementary file 1 [file Data_Sheet_1.doc]

# Supplementary Data

[**Supplementary Table 1 The Final Model and Model-fit Statistics in Women and Men** 2](#__RefHeading___Toc121158128)

[**Supplementary Table 2 Description of the missing data** 3](#__RefHeading___Toc121158129)

[**Supplementary Table 3 Imputed Baseline Characteristics of the Study Population After Imputation** 5](#__RefHeading___Toc121158130)

[**Supplementary Table 4 HRs (95% CIs) of Retinal Arteriosclerosis Development According to SUA Trajectory Groups in Total Population** 6](#__RefHeading___Toc121158131)

[**Supplementary Table 5 Subgroup Analysis of Incidence of Retinal Arteriosclerosis According to SUA Trajectory Groups** 7](#__RefHeading___Toc121158132)

[**Supplementary Figure 1 BIC and AIC in models with different numbers of trajectories in (A) women and (B) men** 8](#__RefHeading___Toc121158133)

[**Supplementary Figure 2 Fitted trajectories of serum uric acid over 5 years (exposure period) in total population** 9](#__RefHeading___Toc121158134)

[**Supplementary Figure 3 Adjusted HRs (95% CIs) of retinal arteriosclerosis development in women subgroup analysis** 10](#__RefHeading___Toc121158135)

[Supplementary Figure 4 Adjusted HRs (95% CIs) of retinal arteriosclerosis development in men subgroup analysis 11](#__RefHeading___Toc121158136)

## Supplementary Table 1 The Final Model and Model-fit Statistics in Women and Men

| **No. of trajectories** | **Parameters of trajectory shape** ^*^ | **Allocated group membership (%)** | **Estimated group membership (%)** | **AvPP** |
| --- | --- | --- | --- | --- |
| Women |  | 17.65 | 293 | 0.86 |
| 4 | 1 | 44.89 | 838 | 0.84 |
|  | 1 | 30.53 | 529 | 0.88 |
|  | 1 | 6.93 | 121 | 0.91 |
|  | 1 |  |  |  |
| Men |  |  |  |  |
| 4 | 1 | 16.35 | 408 | 0.89 |
|  | 1 | 45.01 | 1163 | 0.89 |
|  | 1 | 32.19 | 813 | 0.90 |
|  | 1 | 6.45 | 159 | 0.92 |

^*^ Presented as linear = 1, quadratic = 2, cubic = 3.

AvPP, average posterior probability.

## Supplementary Table 2 Description of the missing data

|  | **Study Population (n=4324)** | |
| --- | --- | --- |
|  | **Before multiple imputation** | **Missing values, n (%)** |
| Age (years) | 46.0 (40.0, 51.0) | 0 (0) |
| Sex (%) |  | 0 (0) |
| Male | 2543 (58.8) |  |
| Female | 1781 (41.2) |  |
| BMI (kg/m^2^) | 23.28 (21.36, 25.24) | 0 (0) |
| SBP (mmHg) | 110.0 (100.0, 120.0) | 30 (0.7) |
| DBP (mmHg) | 70.0 (65.0, 75.0) | 29 (0.7) |
| Smoking status (%) |  | 642 (14.8) |
| Current | 2344 (63.7) |  |
| Former or never | 1338 (36.3) |  |
| Alcohol drinking status (%) |  | 640 (14.8) |
| Current | 1606 (43.6) |  |
| Former or never | 2078 (56.4) |  |
| CVD (%) |  | 0 (0) |
| Positive | 29 (0.7) |  |
| Negative | 4295 (99.3) |  |
| Fasting blood concentrations of: |  |  |
| [FBG (mmol/L)](javascript:;) | 5.21 (4.96, 5.49) | 0 (0) |
| TG (mmol/L) | 1.15 (0.78, 1.71) | 0 (0) |
| TC (mmol/L) | 4.81 (4.26, 5.37) | 0 (0) |
| LDL-C (mmol/L) | 3.10 (2.59, 3.60) | 0 (0) |
| HDL-C (mmol/L) | 1.36 (1.14, 1.63) | 0 (0) |
| Cr (μmol/L) | 68.25 (57.10, 77.90) | 0 (0) |
| eGFR (mL/min/1.73 m^2^) | 98.68 (81.66, 150.84) | 0 (0) |
| SUA (μmol/L) | 319.90 (263.08, 380.42) | 0 (0) |

Continuous variables are expressed as median (interquartile range). Categorical variables are expressed as frequency (percent), the denominators for proportions are the totals at the top of the columns.

BMI, body mass index; SBP, systolic blood pressure; DBP, diastolic blood pressure; CVD, cardiovascular disease; FBG, fasting blood glucose; TG, triglycerides; TC, total cholesterol; LDL-C, low density lipoprotein; HDL-C, high density lipoprotein; Cr, creatinine; eGFR, estimated glomerular filtration rate; SUA, serum uric acid.

## Supplementary Table 3 Imputed Baseline Characteristics of the Study Population After Imputation

|  | **After imputation**  **(the first dataset)** | **After imputation**  **(the second dataset)** | **After imputation**  **(the third dataset)** | **After imputation**  **(the fourth dataset)** | **After imputation**  **(the fifth dataset)** |
| --- | --- | --- | --- | --- | --- |
| Smoking status (%) |  |  |  |  |  |
| Current | 1352 (31.3) | 1353 (31.3) | 1355 (31.3) | 1354 (31.3) | 1360 (31.5) |
| Former or never | 2972 (68.7) | 2971 (68.7) | 2969 (68.7) | 2970 (68.7) | 2964 (68.5) |
| Alcohol drinking status (%) |  |  |  |  |  |
| Current | 2168 (50.1) | 2184 (50.5) | 2194 (50.7) | 2196 (50.8) | 2203 (50.9) |
| Former or never | 2156 (49.9) | 2140 (49.5) | 2130 (49.3) | 2128 (49.2) | 2121 (49.1) |
| SBP (mmHg) | 110.0 (100.0, 120.0) | 110.0 (100.0, 120.0) | 110.0 (100.0, 120.0) | 110.0 (100.0, 120.0) | 110.0 (100.0, 120.0) |
| DBP (mmHg) | 70.0 (65.0, 75.0) | 70.0 (65.0, 75.0) | 70.0 (65.0, 75.0) | 70.00 (65.0, 75.0) | 70.0 (65.0, 75.0) |

Continuous variables are expressed as median (interquartile range). Categorical variables are expressed as frequency (percent).

SBP, systolic blood pressure; DBP, diastolic blood pressure.

## Supplementary Table 4 HRs (95% CIs) of Retinal Arteriosclerosis Development According to SUA Trajectory Groups in Total Population

|  | **Model 1** | | **Model 2** | |
| --- | --- | --- | --- | --- |
| **Variables** | **HR, (95%CI)** | ***p*** **Value** | **HR, (95%CI)** | ***p*** **Value** |
| Total population |  |  |  |  |
| Low | 1.00 (ref) | - | 1.00 (ref) | - |
| Moderate | 1.37(0.99,1.89) | 0.0583 | 0.77(0.54,1.10) | 0.1533 |
| Moderate-high | 2.30(1.70,3.11) | <0.0001 | 0.97(0.65,1.44) | 0.8879 |
| High | 3.57(2.53,5.03) | <0.0001 | 1.30(0.83,2.05) | 0.2510 |
| *p* for trend ^*^ |  | <0.0001 |  | 0.0417 |

Model 1：not adjust.

Model 2：adjustment for age, body mass index, sex, systolic blood pressure, diastolic blood pressure, alcohol drinking status (in men and total-people), triglyceride level, total cholesterol, low-density lipoprotein cholesterol level, high-density lipoprotein cholesterol level, creatinine, and estimated glomerular filtration rate at baseline (from June 1, 2010, through June 1, 2011).

ref: reference.

^*^ Tests of trend were conducted by assessing the statistical significance across categorical SUA trajectory groups as an ordinal variable.

## Supplementary Table 5 Subgroup Analysis of Incidence of Retinal Arteriosclerosis According to SUA Trajectory Groups

|  |  | | **SUA trajectory group** | | | | | | | |  |
| --- | --- | --- | --- | --- | --- | --- | --- | --- | --- | --- | --- |
|  | **All** | | **Low** | | **Moderate** | | **Moderate-high** | | **High** | |  |
| **Subgroups** | **Total, n** | **Incidence, n (ID** ^*^**)** | **Total, n** | **Incidence, n (ID** ^*^**)** | **Total, n** | **Incidence, n (ID** ^*^**)** | **Total, n** | **Incidence, n (ID** ^*^**)** | **Total, n** | **Incidence, n (ID** ^*^**)** | ***p* for trend** ^#^ |
| Women |  |  |  |  |  |  |  |  |  |  |  |
| Age(year) |  |  |  |  |  |  |  |  |  |  |  |
| Age < 45 | 769 | 9(1.2) | 130 | 4(3.3) | 400 | 3(0.8) | 208 | 2(1.0) | 31 | 0(0) | 0.1054 |
| Age ≥45 | 1012 | 88(9.5) | 163 | 9(5.9) | 438 | 35(8.7) | 321 | 31(10.6) | 90 | 13(16.2) | 0.0146 |
| BMI (kg/m^2^) |  |  |  |  |  |  |  |  |  |  |  |
| BMI < 24 | 1416 | 54(4.1) | 261 | 11(4.5) | 706 | 25(3.8) | 384 | 13(3.6) | 65 | 5(8.4) | 0.6754 |
| BMI ≥24 | 365 | 43(13.0) | 32 | 2(6.8) | 132 | 13(10.8) | 145 | 20(15.4) | 56 | 8(16.0) | 0.1512 |
| SBP (mmHg) |  |  |  |  |  |  |  |  |  |  |  |
| SBP<120 | 1529 | 66(4.6) | 258 | 8(3.3) | 738 | 31(4.5) | 443 | 22(5.4) | 90 | 5(6.0) | 0.1936 |
| SBP≥120 | 252 | 31(13.6) | 35 | 5(15.6) | 100 | 7(7.6) | 86 | 11(14.1) | 31 | 8(31.5) | 0.0769 |
| Men |  |  |  |  |  |  |  |  |  |  |  |
| Age(year) |  |  |  |  |  |  |  |  |  |  |  |
| Age < 45 | 1059 | 34(3.4) | 150 | 2(1.4) | 500 | 15(3.2) | 341 | 14(4.4) | 68 | 3(4.8) | 0.0982 |
| Age ≥45 | 1484 | 261(19.9) | 258 | 29(12.5) | 663 | 100(16.9) | 472 | 111(27.2) | 91 | 21(26.4) | <0.0001 |
| BMI (kg/m^2^) |  |  |  |  |  |  |  |  |  |  |  |
| BMI < 24 | 1155 | 85(8.0) | 271 | 20(8.0) | 579 | 35(6.5) | 263 | 26(10.9) | 42 | 4(10.3) | 0.2226 |
| BMI ≥24 | 1388 | 210(16.9) | 137 | 11(8.8) | 584 | 80(15.2) | 550 | 99(20.3) | 117 | 20(19.3) | 0.0040 |
| SBP (mmHg) |  |  |  |  |  |  |  |  |  |  |  |
| SBP<120 | 1566 | 149(10.4) | 277 | 21(8.3) | 744 | 63(9.2) | 465 | 55(13.0) | 80 | 10(13.6) | 0.0233 |
| SBP≥120 | 977 | 146(16.7) | 131 | 10(8.3) | 419 | 52(13.7) | 348 | 70(23.0) | 79 | 14(20.3) | 0.0004 |

^*^ Incidence density per 1000 person-years. ^#^ Tests of trend were conducted by the Cochran–Armitage test.

## Supplementary Figure 1 BIC and AIC in models with different numbers of trajectories in (A) women and (B) men


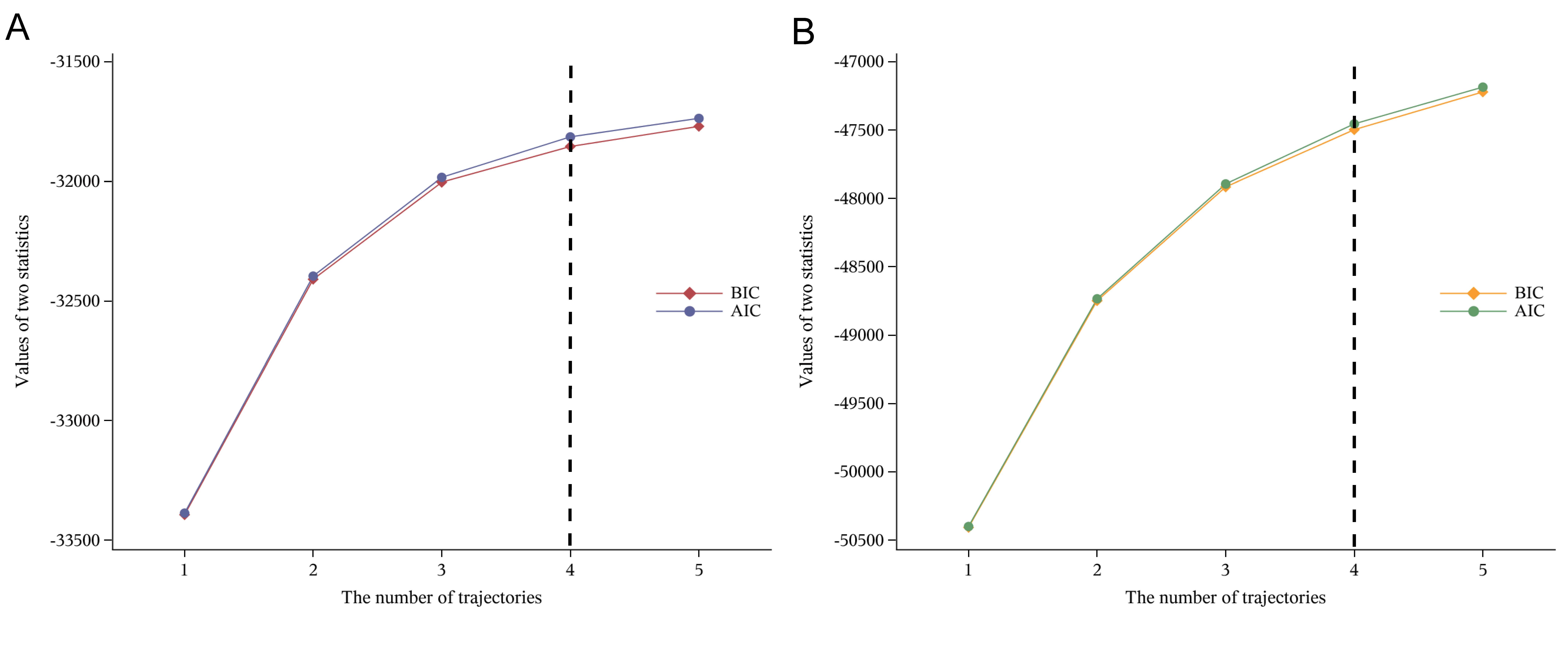


BIC: Bayesian information criterion; AIC: Akaike information criterion.

## Supplementary Figure 2 Fitted trajectories of serum uric acid over 5 years (exposure period) in total population


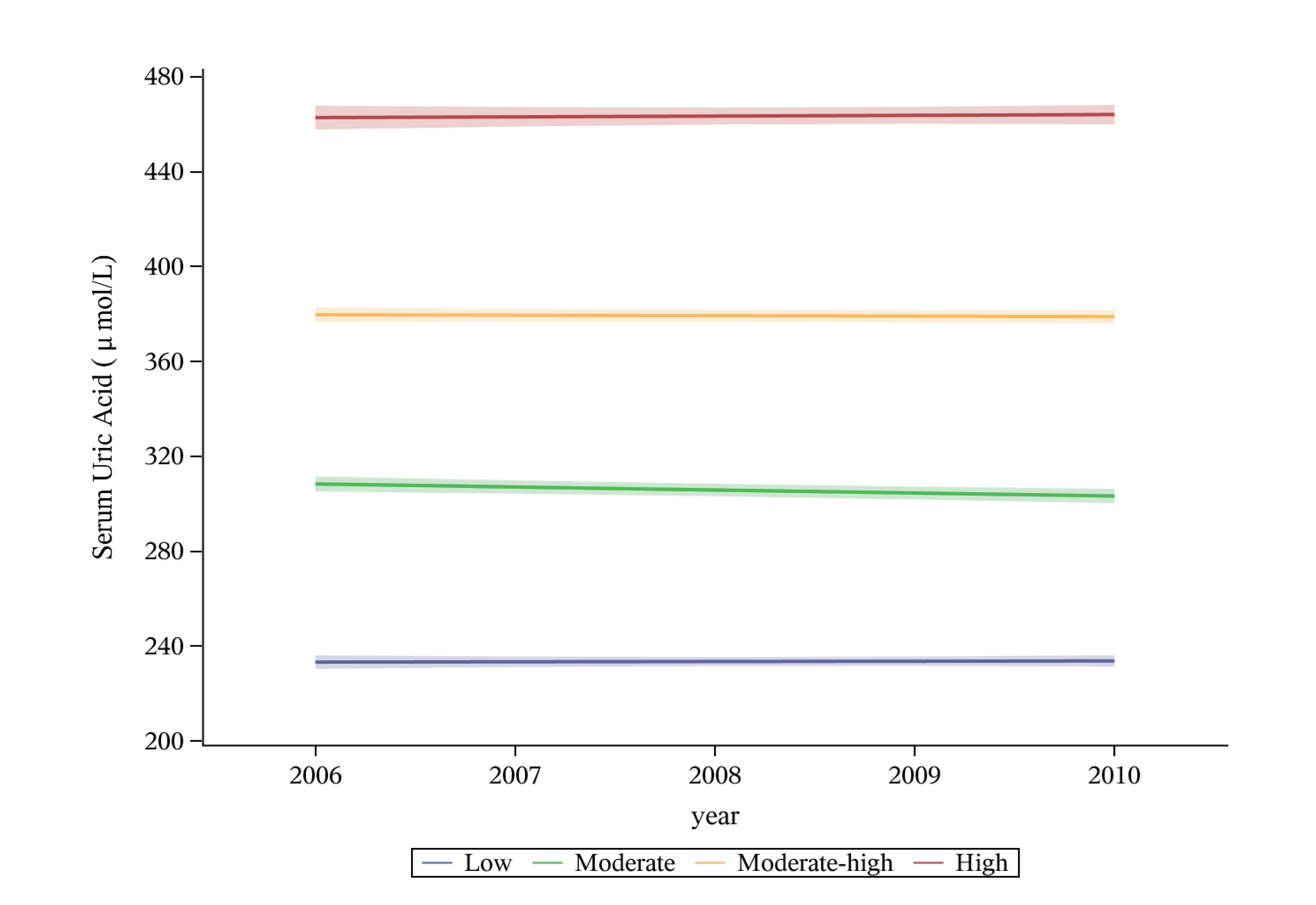


## Supplementary Figure 3 Adjusted HRs (95% CIs) of retinal arteriosclerosis development in women subgroup analysis





The model was adjusted for age, body mass index, systolic blood pressure, diastolic blood pressure, triglyceride level, total cholesterol, low-density lipoprotein cholesterol level, high-density lipoprotein cholesterol level, creatinine, and estimated glomerular filtration rate at baseline (from June 1, 2010, through June 1, 2011).

## Supplementary Figure 4 Adjusted HRs (95% CIs) of retinal arteriosclerosis development in men subgroup analysis





The model was adjusted for age, body mass index, systolic blood pressure, diastolic blood pressure, alcohol drinking status, triglyceride level, total cholesterol, low-density lipoprotein cholesterol level, high-density lipoprotein cholesterol level, creatinine, and estimated glomerular filtration rate at baseline (from June 1, 2010, through June 1, 2011).
